# Large improvement of mental health during in outpatient short-term group psychotherapy treatment—a naturalistic pre-/post-observational study

**DOI:** 10.1007/s40211-022-00449-6

**Published:** 2022-12-08

**Authors:** David Riedl, Karin Labek, Ines Gstrein, Maria-Sophie Rothmund, Barbara Sperner-Unterweger, Wilhelm Kantner-Rumplmair

**Affiliations:** 1 University Clinic of Psychiatry II, Department of Psychiatry, Psychotherapy Psychosomatics and Medical Psychology, Medical University of Innsbruck, Anichstr. 35, 6020 Innsbruck, Austria; 2 Ludwig Boltzmann Institute for Rehabilitation Research, Vienna, Austria; 3 Institute of Psychology, University of Innsbruck, Innsbruck, Austria; 4 Tyrolean Regional Association of Psychotherapy, Innsbruck, Austria

**Keywords:** Depression, Anxiety, Effectiveness, Work ability index, Group treatment, Psychological distress, Depression, Angst, Wirksamkeit, Arbeitsfähigkeitsindex, Gruppenbehandlung, Psychische Belastung

## Abstract

**Background:**

Group psychotherapy is an effective treatment for patients with mental health issues. This study aims to evaluate data on the effectiveness of a cost-free short-term outpatient group psychotherapy project for patients with mixed mental health issues in Tyrol, Austria.

**Methods:**

In this naturalistic observational study, outpatients taking part in the psychotherapeutic group treatment between spring 2018 and spring 2020 were included. Patients completed the patient health questionnaire (PHQ-D), an item of the working ability index (WAI) and single items on symptom burden, treatment expectation and perceived benefit before the first (T0) and/or last group session (T1). Mean changes were investigated using repeated measure analyses of variance (rANOVA).

**Results:**

A total of 98 patients were included in the study. Statistically significant improvements with medium to large effect sizes were observed for depression (η^2^ = 0.22, *p* < 0.001), somatization (η^2^ = 0.10, *p* = 0.008), anxiety (η^2^ = 0.18, *p* < 0.001), and subjective working ability (η^2^ = 0.22, *p* < 0.001). Neither age (*p* = 0.85), sex (*p* = 0.34), baseline symptoms (*p* = 0.29–0.77), nor previous experience with individual (*p* = 0.15) or group psychotherapy (*p* = 0.29) were associated with treatment outcome. However, treatment expectation at baseline was significantly associated with the patients’ perception of the treatment benefit (r = 0.39, *p* < 0.001).

**Conclusion:**

Our study highlights the benefit of outpatient short-term group psychotherapy for individuals with mental health issues. Group psychotherapy should be offered free of charge to individuals with mental health issues by social health providers.

## Abstract (de)

**Hintergrund:**

Gruppenpsychotherapie ist eine wirksame Behandlung für Patienten mit psychischen Problemen. Ziel dieser Studie ist es, Daten über die Wirksamkeit eines kostenfreien ambulanten Kurzzeit-Gruppenpsychotherapieprojekts für Patienten mit verschiedenen psychischen Problemen in Tirol (Österreich) darzustellen.

**Methoden:**

In diese naturalistische Beobachtungsstudie wurden ambulante Patientinnen und Patienten eingeschlossen, die zwischen Frühjahr 2018 und Frühjahr 2020 an der Gruppenpsychotherapie teilnahmen. Die PatientInnen bearbeiteten den Patient Health Questionnaire (PHQ-D), ein Item des Arbeitsfähigkeitsindex (WAI) und zusätzliche Items zur Symptombelastung, zur Wirksamkeitserwartung und zum wahrgenommenen Nutzen vor der ersten (T0) und/oder letzten Gruppensitzung (T1). Mittlere Veränderungen wurden mithilfe von Varianzanalysen mit Messwiederholungen (rANOVA) untersucht.

**Ergebnisse:**

Insgesamt wurden *n* = 98 Patienten in die Studie aufgenommen. Statistisch signifikante Verbesserungen mit mittelgradigen bis großen Effektstärken wurden für Depression (η^2^ = 0,22, *p* < 0,001), Somatisierung (η^2^ = 0,10, *p* = 0,008), Angst (η^2^ = 0,18, *p* < 0,001) und subjektive Arbeitsfähigkeit (η^2^ = 0,22, *p* < 0,001) beobachtet. Weder Alter (*p* = 0,85), Geschlecht (*p* = 0,34) oder Symptombelastung zu Beginn der Behandlung (*p* = 0,29–0,77) noch frühere Erfahrungen mit Einzel- (*p* = 0,15) oder Gruppenpsychotherapie (*p* = 0,29) waren mit dem Behandlungsergebnis assoziiert. Allerdings zeigte sich ein signifikanter Zusammenhang zwischen der Wirksamkeitserwartung zu Beginn und dem wahrgenommenen Nutzen beim Abschluss der Therapie (r = 0,39, *p* < 0,001).

**Fazit:**

Unsere Studie unterstreicht den Nutzen ambulanter Kurzzeit-Gruppenpsychotherapie für Menschen mit psychischen Problemen. Menschen mit psychischen Problemen ein sollte von Sozialversicherungsträgern eine kostenfreie Gruppenpsychotherapie angeboten werden.

## Background

One of the most critical current public health challenges is to provide mental health interventions in ways that are effective, affordable, and have the potential for large-scale applications. The current World Health Organization “Mental Health Action Plan 2013–2030” highlights the central role of mental health in achieving health and equity for all people by providing universal health coverage. This agenda has to compete with high prevalence rates. For example, in EU countries, the latest estimates of the prevalence of mental disorders indicated having 17% of EU citizens suffer from mental disorders each year [1]. A recently published epidemiological survey of the adult population in Austria reported a 4.3–9.9% (2-week prevalence) and 15.6% (10-year prevalence) for depression, and 6.5% of anxiety disorders [2, 3]. In addition to the suffering mental impairment causes for those affected and their families, it also imposes economic and societal burden. It was estimated that in 2010 a total of up to 8.5 trillion US dollars in lost output could be attributed to mental health issues [4] and that anxiety and depression alone lead to a worldwide loss of more than 12 billion days of productivity each year [5]. A study published in 2020 reported 37% higher healthcare costs and twice as high total costs among individuals with major depressive disorder (MDD) compared to individuals without MDD in Austria, and lost productivity costs over 2.5-times higher for those with MDDs [6].

Psychotherapy has become a widely approved treatment intervention for mental disorders [7] and research in recent years has demonstrated the efficacy and efficiency of psychotherapy in multiple studies [8, 9]. The National Institute for Health and Care Excellence (NICE) guidelines consider psychotherapy even as less intrusive than psychopharmaceuticals and, thus, recommend it as the treatment of choice for anxiety disorders [10]. As for depression in outpatients, psychotherapy and pharmacotherapy were found to be equally efficacious in the short term, while psychotherapy showed superior outcomes in the long term [11]. Thus, psychotherapy is considered a key element in the treatment of mental health issues and is recommended as treatment in several treatment guidelines [12, 13].

Psychotherapeutic treatment usually consists of individual treatment based on cognitive–behavioral, interpersonal or psychodynamic approaches. Besides individual psychotherapy, several conceptualizations and manuals for group therapies have been developed over the last few decades. Psychotherapeutic group therapy usually includes six to twelve participants and either one or two psychotherapists. The groups can either have a closed format, i.e., a fixed number of participants and sessions or an open format, i.e., and ongoing group where patients can join at predefined timepoints [14]. Despite organizational challenges around group therapy, such as a difficulty to find appropriate rooms or, especially in rural areas, of finding a sufficient number of patients who want to participate, group psychotherapy has several advantages over individual therapy. For one, patients often find a sense of cohesion when sharing their experiences with others who are facing similar problems. In addition, the group setting offers more interpersonal feedback and allows an easier understanding of the relational problems patients often have to face outside the therapy setting too [14]. Research has shown that group therapy is an effective treatment for different mental health issues, including anxiety and depression [15–17]. A recent review suggested that group psychotherapy was equally effective in reducing a patient’s psychological distress and symptoms than individual psychotherapy [18].

In Austria, group therapy is most frequently applied in inpatient settings, e.g., inpatient psychotherapy wards or inpatient rehabilitation centers, while outpatients usually receive psychotherapy in the individual setting. In contrast to Germany, psychotherapy is usually not covered by the general health insurance in Austria [19]. However, the local health insurance providers in each federal state have developed financial support systems for patients in their region. As these models are largely underfunded, patients have long waiting periods or must pay for their psychotherapy out of pocket [20]. A recent study of > 6000 patients from a psychosomatic rehabilitation center in Austria showed that less than half of the patients had received funding from their health insurance. The majority of patients who self-financed psychotherapy paid more than 50–100 € per session, while a third of this sample earned less than 1000 € a month [20]. This clearly shows that psychotherapy is still a scarce good for many patients in need in Austria. To meet this demand, the regional health care provider of the federal state of Tyrol (ÖGK Tirol) launched a pilot project for cost-free group therapy in association with the Tyrolean Regional Association of Psychotherapy (TLP) starting in spring 2018. The present study aims to provide data on the effectiveness of the group therapy project in improving mental health of participating patients.

## Methods

### Sample and procedure

This longitudinal observational study included outpatients participating in psychotherapeutic group treatment offered by the Tyrolean Regional Association of Psychotherapy between spring 2018 and spring 2020. Each group included a heterogeneous population of patients with different diagnoses and various mental health problems. To take part in the treatment, patients had to be insured by the ÖGK and had to be assigned to the treatment by a physician. Patients completed questionnaires before the first (T0) and last group session (T1). The patient identity was fully anonymized, i.e., patients chose an individual code to match pre- and posttreatment questionnaires, which was unknown to the psychotherapists and researchers, and patients signed a written informed consent prior to study inclusion. The study design was in accordance with the Declaration of Helsinki (1964) and its later amendments and was approved by the research ethics committee of the University of Innsbruck (No. 63/2022).

### Recruitment procedure and psychotherapeutic treatment

The group therapy was advertised on the homepage of the TLP, in local newspapers, and by sending out information to regional hospitals with psychotherapeutic in- or outpatient wards. To facilitate access to the treatment, psychotherapeutic groups were offered in five regions of Tyrol (Oberland, Unterland, Außerfern, Innsbruck Stadt, Reutte). Psychotherapeutic treatment consisted of 10 or 20 weekly or bi-weekly group sessions with a duration of 90 min each, adding up to a treatment duration between 3 and 6 months. The treatment was fully financed by the ÖGK as part of the Tyrolean Group Psychotherapy Pilot Project. Treatment consisted of closed groups, guided by two psychotherapists with experience in group therapy and different therapeutic backgrounds (i.e., cognitive–behavioral, psychoanalytic/psychodynamic, humanistic or systemic^1^). Each group was guided by two therapists, usually with therapists from different treatment methods. Groups were either offered as large groups (i.e., up to 12 participants with 20 group sessions) or small groups (6–8 participants with 10 group sessions). Patients could register for group therapy on the TLP homepage and were then invited to a first consultation free of cost with one of the therapists of the group they registered for. In this meeting, patients were informed about treatment modalities, organizational details, and the evaluation study, and it was evaluated whether patients were suited to take part in group psychotherapy.

### Measures

The *Patient Health Questionnaire (PHQ-D)* is a self-rated questionnaire to assess the severity of different mental disorders. The PHQ‑D is a broadly used and well-validated questionnaire which allows categorical scoring for DSM-IV categories. It consists of 78 items to assess several submodules on somatoform (PHQ-15), depressive (PHQ-9), anxiety (PHQ-anx), and eating disorders (PHQ-eat) as well as alcohol abuse (PHQ-subst). Items can be answered on two- to four-point scales, depending on the module. The items allow a categorical scoring for DSM-IV categories of major depression, other depressive disorders, panic disorder, other anxiety disorders, bulimia nervosa, and the alcohol (abuse) syndrome. In addition, severity scores for depression (9 items), somatization (15 items), and anxiety (7 items) can be calculated, with higher scores indicating higher levels of distress. The PHQ‑9 total score ranges from 0 to 27 points, with scores of < 5 points indicating no depression, 5–9 points mild, 10–15 moderate, and ≥ severe levels of depression. Good psychometric properties have been reported for the PHQ‑D [21].

Patients were asked to rate their perceived *working ability* with a single item taken from the Work Ability Index (WAI) [22]. The item could be rated from 0 (‘very poor working ability’) to 4 (‘very good working ability’). The WAI is a broadly used questionnaire to assess working ability in different fields of research. Good psychometric properties have been reported for the WAI [22].

In addition, three additional questions which were specifically designed for this study were applied. Patients were asked to rate their *symptom burden* by indicating how much they suffered from their symptoms in the last week on a scale from 0 (‘not at all’) to 3 (‘very much’). At baseline, *treatment expectation* was evaluated using a single item, asking patients how much they think they will benefit from the intervention from 0 (‘not at all’) to 3 (‘very much’). After treatment patients were asked to rate the *perceived benefit*, i.e., if they (a) reached their goals in therapy, (b) perceived the group as helpful, (c) perceived the psychotherapists as helpful, and (d) if they would recommend the group therapy to significant others on a scale from 0 (‘not at all’) to 3 (‘very much’).

### Statistical procedures

Symptom improvement was evaluated using repeated measures analysis of variance (rANOVAs), with pre- and posttreatment PHQ‑D depression, anxiety, and somatization scores as within-subject factors. For sensitivity analyses, calculations were repeated with group size and duration (small group with 10 sessions vs. large group with 20 sessions) as a between-subject factor to determine whether the group size and therapy duration influenced the outcome.

Univariate analyses were conducted to investigate which baseline variables are associated with the patients evaluation if they had reached their treatment goal after treatment, including *sociodemographic variables* (age, sex, relationship status, living situation), *treatment-related factors* (group assignment, psychiatric diagnosis, psychiatric medication, previous experience with individual or group therapy), *symptoms* (PHQ depression, anxiety and somatization scores at baseline), as well as *treatment expectation*.

Effect sizes of *η*^*2*^ = 0.01 were considered small, while *η*^*2*^ = 0.06 indicated a medium, and *η*^*2*^ = 0.14 a large effect, respectively. For 2 × 2 contingency tables ϕ‑values (0.1 = small, 0.3 = medium, and 0.5 = large effects) are reported [23].

## Results

A total of 98 patients completed the questionnaires at T0 and were thus included in the study. A mean number of 8 patients (range 5–12 patients) per group participated in therapy. The vast majority of patients were female (> 70%), between 40–60 years, and living with their partner and/or children. Most patients were assigned to the group psychotherapy treatment by a psychotherapist (*n* = 37; 37.8%), a hospital (*n* = 21; 21.4%), or a psychiatrist (*n* = 19; 19.4%) and about two thirds had a pre-existing psychiatric diagnosis (*n* = 67; 68.4%). While 70 (71.4%) patients had experiences with individual psychotherapy, only 39 (39.8%) had previously taken part in group psychotherapy. For details see Table 1.

**Table 1:** Sociodemographic and clinical characteristics

	*N*	%
*Sex*		
Male	26	26.5
Female	72	73.5
*Age *(M = 45.6; SD = 12.5)		
< 40	34	34.7
40–60	52	53.1
> 60	12	12.2
*Relationship*		
Married	39	39.8
Single	28	28.6
Divorced	23	23.5
Other	7	7.1
Missing	1	1.0
*Living situation*		
Alone	37	37.8
With partner/children	49	50.0
With family of origin	7	7.1
In a shared flat	3	3.1
Missing	2	2.0
*Assigned to group therapy by*		
Health insurance provider	2	2.0
Hospital	21	21.4
Psychotherapist	37	37.8
General practitioner	5	5.1
Psychiatrist	19	19.4
Other	11	11.2
Missing	3	3.1
*Psychiatric diagnosis*	67	68.4
Missing	10	10.2
*Currently with psychiatric medication*	71	72.4
Missing	2	2.0
*Previous individual psychotherapy*	70	71.4
Missing	9	9.2
*Previous group psychotherapy*	39	39.8
Missing	10	10.2
*Previous psychotherapeutic inpatient treatment*	32	32.7
Missing	10	10.2

*SD* standard deviation, *M* mean

### Proportion of patients meeting diagnostic criteria before and after group psychotherapy

At baseline, patients most frequently fulfilled the criteria for depressive disorders (58.8%), followed by somatization disorder (48.0%), and problematic alcohol consumption (44.9%). The vast majority (*n* = 66, 67.4%) fulfilled criteria for more than one diagnosis, with a median number of two diagnoses per patient. Prevalence for all assessed syndromes, apart from Bulimia nervosa, was significantly lower after therapy in comparison to baseline. For details see Table 2.

**Table 2:** Proportion of patients meeting diagnostic criteria based on the PHQ‑D patient self-assessment before (T0) and after (T1) the intervention

	T0 (*n* = 98)		T1 (*n* = 80)			
	n	%	n	%	*χ*^*2*^	*p*-value
MDD	23	23.5	13	16.3	6.66	0.010
Other depressive disorders	57	58.8	23	28.8	8.03	0.005
Somatization	47	48.0	22	27.5	12.41	< 0.001
Anxiety	36	36.7	16	20.0	5.34	0.021
Panic	25	25.5	16	20.0	17.03	< 0.001
Bulimia nervosa	2	2.0	4	5.0	9.99	0.09
Alcohol syndrome	44	44.9	34	42.5	19.87	< 0.001

*MDD* major depressive disorder

### Severity of depression, anxiety, and somatization levels before and after group psychotherapy

**Fig. 1:**
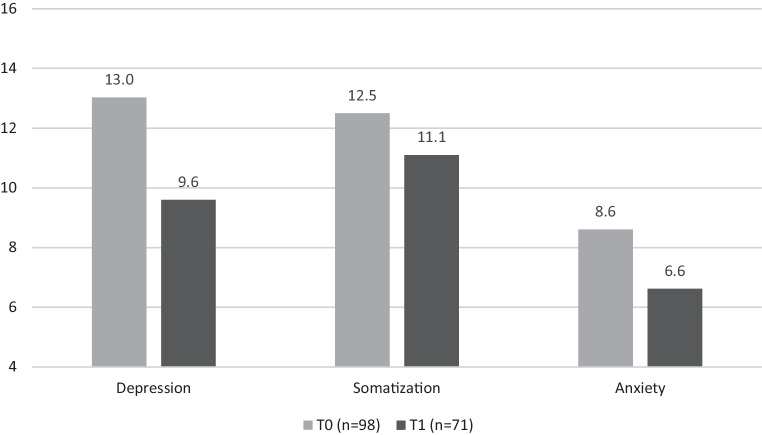
Effect sizes for pre- (T0) and posttreatment (T1) PHQ‑D scores for symptoms of depression, anxiety, and somatization with effect sizes

To analyze the improvement of mental health issues, repeated measures ANOVAs were calculated for depression, somatization, and anxiety severity scores before and after intervention. Statistically significant improvement with large effect sizes was found for depression (F (1,70) = 15.82, *p* < 0.001, η^2^ = 0.18), somatization (F (1,69) = 7.53, *p* = 0.008, η^2^ = 0.10), and anxiety (F (1,69) = 16.31, *p* < 0.001, η^2^ = 0.19). Neither group size nor sex influenced the outcome, neither for depression (*p* = 0.41; *p* = 0.32), anxiety (*p* = 0.24; *p* = 0.68), or somatization (*p* = 0.84; *p* = 0.14). Details are shown in Fig. 1.

**Fig. 2:**
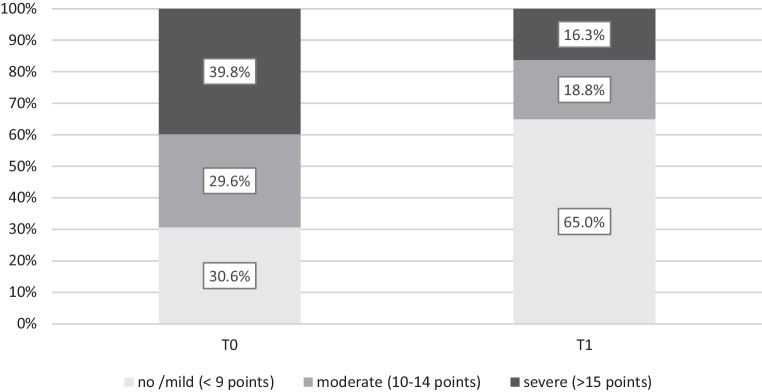
Severity of depressive symptoms before (T0) and after (T1) group psychotherapy based on the PHQ‑9 total scores

The most pronounced improvements were found for depression with a mean symptom reduction of 3.4 points. When comparing the severity levels of depression before and after treatment, a clear pattern of symptom improvement could be observed. While almost 70% of the sample reported moderate to severe depressive symptoms before treatment, the majority of patients (65%) reported no or only mild symptoms after the treatment. For details see Fig. 2.

### Symptom burden and working ability before and after group psychotherapy

**Fig. 3:**
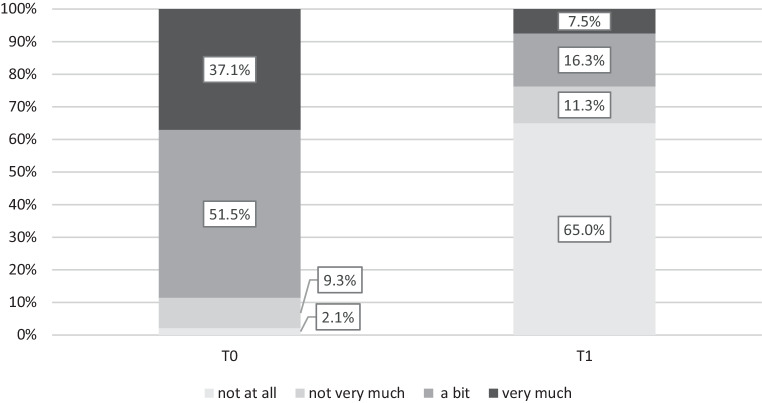
Symptom burden as reported by patients before (T0) and after (T1) group psychotherapy

A clear trend was observed in the symptom burden as rated by the patients. Before treatment, the vast majority of patients (88.6%) had reported having suffered at least a bit from their symptoms. This number significantly decreased (23.8%) after treatment, with most patients (65.0%) reporting that they did not suffer at all from their symptoms. This improvement was statistically significant with a large effect size (F (1,76) = 141.05, *p* < 0.001; η^2^ = 0.65). For details, see Fig. 3.

Similar results were observed for the patient-reported working ability. The mean scored working ability significantly improved during the psychotherapy with a large effect size (F (1,82) = 22.83, *p* < 0.001; η^2^ = 0.22). Before treatment, 67% of the patients rated their working ability as very poor or poor, 22.4% as moderate and 9.2% as good or very good. After treatment this rate improved with 24.0% of the patients rating their working ability as good or very good, 25.3% as moderate and 50.7% as poor or very poor.

### Perceived benefit of the group psychotherapy

The vast majority of patients reported achieving their goals as a result of the intervention (76.3%), regardless of age (*p* = 0.85), sex (*p* = 0.34), baseline depression (*p* = 0.29), anxiety (*p* = 0.77), or somatization scores (*p* = 0.51) as well as previous experience with individual (*p* = 0.15) or group psychotherapy (*p* = 0.29). However, higher treatment expectation at baseline was significantly associated with the patient’s evaluation of reaching their treatment goals (r = 0.39, *p* < 0.001).

## Discussion

In this naturalistic study based on real-world data, we aimed to investigate to what extent short-term group psychotherapy reduced psychological distress in outpatients with various mental health problems. In line with previous research, we found improvements with large effect sizes for depression, anxiety, and somatization.

At baseline, almost 60% of the patients fulfilled the criteria for a depressive disorder and almost 50% for somatization and alcohol abuse. These findings are consistent with epidemiological research in Austria, which found especially depression and alcohol abuse to be highly prevalent in the general population [3]. Depression is a major health risk and has been associated with decreased quality of life [24] and quality-adjusted life years (QALYs) [25], and even higher mortality [26]. Depression is also strongly linked to substance abuse and drinking patterns [27]. The consumption of alcohol is culturally broadly accepted in Austria, and there is a tendency to belittle the multiple toxic side effects of alcohol abuse. These include an increased risk for physical comorbidities such as infectious diseases, cancer, diabetes or cardiovascular disease, problems in interpersonal relationships and the workplace, lower capacity to deal with aggression, and an increased risk for economic and legal problems [28].

We could observe a substantially reduced level of anxiety, depression, and somatization in our sample by the end of the group therapy. While before treatment, about two-thirds of the sample reported moderate to severe symptoms of depression, by the end of treatment, the rates were inversed, with two-thirds of patients reporting none or only mild symptoms of depression. Our results align with previous research that found large effect sizes for major symptom clusters such as anxiety or depression [18]. In contrast to previous findings [29], group size was not associated with treatment outcome, i.e., indicating that patients in smaller and larger groups benefitted equally from the treatment. Since the treatment duration was slightly longer for larger groups, this could possibly have compensated the group size effect. However, it has to be mentioned that no substantial changes in substance abuse were observed in our sample. A probable explanation might be that substance abuse was often a secondary problem resulting from other issues—such as depression or anxiety—and patients focused on their primary burden in the treatment. It might be worthwhile to offer separate groups addressing substance abuse in the future to achieve better outcomes in this clinical population.

In our sample, a substantially larger proportion of female patients participated in group therapy. This is in line with previous studies which reported gender differences in the prevalence of various mental disorders [30, 31]. In addition, men are less likely to seek psychological help than women and are less open to report problems or feelings of insufficiency than women [32]. Ogrodniczuk and Oliffe [33] point out that men may often face challenges in overcoming their cultural norms that prevent them from revealing themselves, which can lead to isolation, distress, and diminished self-awareness. While this may be especially true for psychotherapeutic groups, the evidence on gender-related differences in uptake of group therapy is scarce and merits further research. In contrast to previous research [34], in our study gender was not associated with therapy outcome, neither for anxiety, depression nor somatization, indicating that both men and women equally profited from group psychotherapy.

Patients also reported a significantly improved subjective working ability. While there is some indication that psychotherapy can lead to long-term improvement of patients’ working ability [35], to our knowledge, no study has investigated this for short-term group psychotherapy. Our results thus indicate that group psychotherapy effectively decreases mental health problems and increases the patient’s quality of life, while being relatively cost effective [15]. While cost-saving should never be the leading argument in patient care, is undeniable an important variable to argue for the implementation of therapeutic interventions into clinical practice.

The study has several *strengths and limitations*. For one, to our knowledge, this is the first study to investigate the improvement in mental health during the group psychotherapies offered in the Tyrolean group psychotherapy project. Another strength of our study is the use of an unbiased naturalistic patient sample, which increases the generalizability of our findings. Since no control group was used in the study, it is unclear to what proportion the observed changes in the mental health variables were caused by spontaneous remission. Also, other factors, which could have influenced the improvement of health were not assessed. However, due to the observed large effect sizes, we consider it fair to assume that our study still indicates a certain level of effectiveness of group psychotherapy. Data collected in the study relied on patient self-assessments, which may have been influenced by memory bias and socially desirable answers. We tried to minimize this risk of bias by using questionnaires with a relatively short recall period (i.e., 1–4 weeks) and to completely anonymize patients’ questionnaires. Also, in this study only short-term outcome data of the group therapy was available. A longitudinal follow-up study is planned to evaluate the long-term effects of this treatment.

## Conclusion

In this naturalistic observational study, large improvements in anxiety, depression, and somatization were observed during a short-term group psychotherapy treatment for patients with mixed mental health issues. Group psychotherapy is an effective and cost-efficient way to improve the quality of life of individuals with mental health issues. Based on our findings, we consider group psychotherapy an essential and valuable treatment and strongly recommend providing free access to group therapy for people with mental health problems.

## Abbreviations

ÖGK Tirol: Österreichische Gesundheitskasse Tirol (regional health care provider of the federal state of Tyrol)
PHQ‑D: Patient health questionnaire
TLP: Tyrolean Regional Association of Psychotherapy
WAI: Working ability index

## Footnotes

1 In Austria there are 23 psychotherapeutic methods that are recognized by the ministry of health, which can be clustered in four groups: cognitive–behavioral, psychoanalytic/psychodynamic, humanistic and systemic psychotherapy: https://www.psychotherapie.at/patientinnen/ueber-psychotherapie.

## References

[1] OECD, Union E. Health at a Glance: Europe 2018.

[2] Arias-de la Torre J, Vilagut G, Ronaldson A, Serrano-Blanco A, Martín V, Peters M (2021). Prevalence and variability of current depressive disorder in 27 European countries: a population-based study. Lancet Public Health.

[3] Łaszewska A, Österle A, Wancata J, Simon J (2018). Prevalence of mental diseases in Austria : systematic review of the published evidence. Wien Klin Wochenschr.

[4] Bloom DE, Cafiero E (2011). The global economic burden of noncommunicable diseases.

[5] Chisholm D, Sweeny K, Sheehan P, Rasmussen B, Smit F, Cuijpers P (2016). Scaling-up treatment of depression and anxiety: a global return on investment analysis. Lancet Psychiatry.

[6] Łaszewska A, Wancata J, Jahn R, Simon J (2020). The excess economic burden of mental disorders: findings from a cross-sectional prevalence survey in Austria. Eur J Health Econ.

[7] Barkham M, Lutz W, Castonguay LG (2021). Bergin and Garfield’s handbook of psychotherapy and behavior change.

[8] Burlingame GM, Gleave R, Erekson D, Nelson PL, Olsen J, Thayer S (2016). Differential effectiveness of group, individual, and conjoint treatments: An archival analysis of OQ-45 change trajectories. Psychother Res.

[9] Shedler J (2010). The efficacy of psychodynamic psychotherapy. Am Psychol.

[10] NICE. Anxiety disorders. 2014. https://www.nice.org.uk/guidance/QS53/chapter/Quality-statement-2-Psychological-interventions. Accessed 10.09.2022.

[11] Leichsenring F, Steinert C, Hoyer J (2016). Psychotherapy versus pharmacotherapy of depression: what’s the evidence?. Z Psychosom Med Psychother.

[12] DGPPN B, KBV, AWMF. Unipolar Depression—S3 treatment guideline 2015. www.depression.versorgungsleitlinien.de. Accessed 10.09.2022.

[13] American Psychological Association. Clinical practice guideline for the treatment of depression across three age cohorts. 2019. https://www.apa.org/depression-guideline. Accessed 10.09.2022.

[14] Yalom ID (2015). Theory and practice of group psychotherapy.

[15] McDermut W, Miller WI, Brown RA (2001). The efficacy of group psychotherapy for depression: a meta-analysis and review of the empirical research. Clin Psychol Pract.

[16] Barkowski S, Schwartze D, Strauss B, Burlingame GM, Rosendahl J (2020). Efficacy of group psychotherapy for anxiety disorders: a systematic review and meta-analysis. Psychother Res.

[17] Blackmore C, Tantam D, Parry G, Chambers E (2012). Report on a systematic review of the efficacy and clinical effectiveness of group analysis and analytic/dynamic group psychotherapy. Group Anal.

[18] Rosendahl J, Alldredge CT, Burlingame GM, Strauss B (2021). Recent developments in group psychotherapy research. Am J Psychother.

[19] Riffer F, Knopp M, Burghardt J, Sprung M (2021). Geschlechtsspezifische Unterschiede in der psychotherapeutischen Versorgung. Psychotherapeut.

[20] Riffer F, Knopp M, Oppenauer C, Sprung M (2017). Psychotherapeutische Versorgung in Österreich: Kassenfinanzierte Psychotherapie für Menschen mit chronisch psychischen Erkrankungen im Jahresvergleich 2017 bis 2020. Psychother Forum.

[21] Loewe B, Spitzer RL, Zipfel S, Herzog W (2002). PHQ-D Health Questionnaire—Manual.

[22] Hasselhorn HM, Freude G (2007). The work ability index—a guideline.

[23] Ellis PD (2010). The essential guide to effect sizes: statistical power, meta-analysis, and the interpretation of research results.

[24] Hohls JK, König HH, Quirke E, Hajek A (2021). Anxiety, depression and quality of life—a systematic review of evidence from longitudinal observational studies. Int J Environ Res Public Health.

[25] Jia H, Lubetkin EI (2017). Incremental decreases in quality-adjusted life years (QALY) associated with higher levels of depressive symptoms for U.S. Adults aged 65 years and older. Health Qual Life Outcomes.

[26] Voshaar ORC, Aprahamian I, Borges MK, van den Brink RHS, Marijnissen RM, Hoogendijk EO (2021). Excess mortality in depressive and anxiety disorders: the Lifelines Cohort Study. Eur Psychiatry.

[27] Kuria MW, Ndetei DM, Obot IS, Khasakhala LI, Bagaka BM, Mbugua MN (2012). The Association between alcohol dependence and depression before and after treatment for alcohol dependence. ISRN Psychiatry.

[28] Levola J, Kaskela T, Holopainen A, Sabariego C, Tourunen J, Cieza A (2014). Psychosocial difficulties in alcohol dependence: a systematic review of activity limitations and participation restrictions. Disabil Rehabil.

[29] McLaughlin SPB, Barkowski S, Burlingame GM, Strauss B, Rosendahl J (2019). Group psychotherapy for borderline personality disorder: a meta-analysis of randomized-controlled trials. Psychotherapy.

[30] Salk RH, Hyde JS, Abramson LY (2017). Gender differences in depression in representative national samples: meta-analyses of diagnoses and symptoms. Psychol Bull.

[31] Riecher-Rössler A (2017). Sex and gender differences in mental disorders. Lancet Psychiatry.

[32] Seidler ZE, Rice SM, Ogrodniczuk JS, Oliffe JL, Dhillon HM (2018). Engaging men in psychological treatment: a scoping review. Am J Mens Health.

[33] Ogrodniczuk JS, Oliffe JL (2009). Grief and groups: considerations for the treatment of depressed men. J Mens Health.

[34] Ogrodniczuk JS, Piper WE, Joyce AS (2004). Differences in men’s and women’s responses to short-term group psychotherapy. Psychother Res.

[35] Joyce AS, Piper WE, Ogrodniczuk JS (2007). Therapeutic Alliance and Cohesion Variables as Predictors of Outcome in Short-Term Group Psychotherapy. Int J Group Psychother.

